# Equal but different: Natural ecotones are dissimilar to anthropic edges

**DOI:** 10.1371/journal.pone.0213008

**Published:** 2019-03-04

**Authors:** Giselle M. Lourenço, Glória R. Soares, Talita P. Santos, Wesley Dáttilo, André V. L. Freitas, Sérvio P. Ribeiro

**Affiliations:** 1 Departamento de Biologia Animal, Instituto de Biologia, Universidade Estadual de Campinas, Campinas, São Paulo, Brazil; 2 Departamento de Biologia Geral, Universidade Federal de Viçosa, Viçosa, Minas Gerais, Brazil; 3 Departamento de Biodiversidade, Evolução e Meio Ambiente, Instituto de Ciências Exatas e Biológicas, Universidade Federal de Ouro Preto, Ouro Preto, Minas Gerais, Brazil; 4 Red de Ecoetología, Instituto de Ecología A.C., Xalapa, Veracruz, Mexico; 5 Museu de Zoologia, Instituto de Biologia, Universidade Estadual de Campinas, Campinas, São Paulo, Brazil; Universita degli Studi di Roma Tor Vergata, ITALY

## Abstract

Increasing deforestation worldwide has expanded the interfaces between fragmented forests and non-forest habitats. Human-made edges are very different from the original forest cover, with different microclimatic conditions. Conversely, the natural transitions (i.e., ecotones) are distinct from human-made forest edges. The human-made forest edges are usually sharp associated with disturbances, with abrupt changes in temperature, humidity, luminosity and wind incidence towards the forest interior. However, the natural forest-lake ecotones, even when abrupt, are composed of a complex vegetal physiognomy, with canopy structures close to the ground level and a composition of herbaceous and arboreal species well adapted to this transition range. In the present study, fruit-feeding butterflies were used as models to investigate whether faunal assemblages in natural ecotones are more similar to the forest interior than to the anthropic edges. Butterflies were sampled monthly over one year in the Rio Doce State Park, Southeastern Brazil, following a standardized design using a total of 90 bait traps, in three different forest habitats (forest interior, forest ecotone and anthropic edges), in both canopy and understory. A total of 11,594 individuals from 98 butterfly species were collected (3,151 individuals from 79 species in the forest interior, 4,321 individuals from 87 species in the ecotone and 4,122 individuals from 83 species in the edge). The results indicated that the butterfly richness and diversity were higher in transition areas (ecotones and edges). The ecotone included a combination of butterfly species from the forest interior and from anthropic edges. However, species composition and dominance in the ecotone were similar to the forest interior in both vertical strata. These results suggest that human made forest edges are quite distinct from ecotones. Moreover, ecotones represent unique habitats accommodating species adapted to distinct ecological conditions, while anthropic edges accommodate only opportunistic species from open areas or upper canopies.

## Introduction

Increasing deforestation worldwide has expanded the interfaces between fragmented forests and non-forest habitats (e.g., croplands, pasture, roads and urban areas), and studies of these interfaces dominates the forest ecology literature [1,2]. Human-made edges are very different from the original forest cover, with different microclimatic conditions, including temperature, humidity, wind speed and the amount of solar radiation that penetrates the habitat [3]. These changes in the microclimate can extend into the forest understory and may extend further when the fragments are small [3]. All these edge effects cause changes in the natural community structure, not only in terms of abundance and diversity, but also in the ecological interactions between organisms [4].

As well as human-made edges, there are many kinds of natural transitions between forests and non-forest habitats, such as interfaces with lakes, rivers, riparian forests, dunes, savannas and grasslands, all falling into the category of “ecotones”. Following Holland [5], ecotones are defined as a “zone of transition between adjacent ecological systems, having a set of characteristics uniquely defined by space and time scales and by the strength of the interactions between adjacent ecological systems”. As natural transition habitats, forest ecotones are distinct from human-made forest edges. The human-made forest edges are usually sharp and associated with disturbances, with abrupt changes in temperature, humidity, luminosity and wind incidence from the forest interior towards the edge [3]. In contrast to human edges, natural forest-lake ecotones, for example, even when abrupt are composed of a complex vegetation physiognomy, with trees growing leaning toward to the open habitat and canopy structures close to the ground level (hereafter “brought low canopy”) [6]. Additionally, the forest-lake ecotone has a composition of herbaceous and arboreal species well adapted to this transition range [6]. In these particular ecotones between forest and lakes, the foliage remains close to the ground and yet presents several characteristics similar to the typical forest upper canopy [6]. For example, canopy leaves present a high degree of sclerophylly and typical morphological attributes resulting from high insolation [7–9]. In addition, rates of leaf respiration are similar to those of emergent trees [7] and the crown architecture is typical of a canopy tree (i.e., trunk-leaf biomass ratio, qualitative data on leaf characteristics and trunk ramifications) [6]. Hence, there are eco-physiological similarities already identified between the upper canopy and the brought low canopy.

The difference in height between the ecotone canopy and the canopy of the forest interior or anthropic edges is remarkable. Knowing that the vertical stratification of organisms and resources is maintained mainly by the height differentiation between strata, as well as the amount of light that arrives in each forest stratum [10], it is expected that this stratification will be lost in the ecotone. However, other studies have shown that, even at a lower height, the forest-lake ecotone presents vast territories of dominant ants such as those in the genus *Azteca*, which is a typical ant distribution pattern of the upper canopies [11,12]. Also, a high number of galls are found in the ecotones, and in wet forests, those are typical of the upper canopy as well [8,13]. On the other hand, investigating organisms that present a consistent vertical stratification distribution, such as butterflies, is an opportunity to understand how the characteristics of each habitat type (e.g., natural or human-made transitions), may influence species distribution in the landscape and ultimately the whole community.

In tropical forests, fruit-feeding butterflies (i.e., those whose adults primarily obtain nutrients by feeding on rotten fruits or fermenting sap; [14]) are considered an excellent model for studies of community structure and temporal variation in diversity. Mainly because fruit-feeding butterflies are ecologically diverse, sensitive to seasons and to habitat quality, have a taxonomy relatively well resolved and are sampled with traps baited with rotting fruits, allowing for simultaneous and standardized sampling in several areas, revised by [15]. In addition, fruit-feeding butterflies respond to the vertical structure of forest and several types of disturbance, which reinforces their role as biological indicators [16–20]. Previous studies have shown that butterflies are highly sensitive to fragmentation and to changes in forest cover [20–26], responding to variation in the immediate surrounding vegetation and to different intensities of disturbance [17]. Less intense land use tends to increase the abundance and richness species, while the more intensive land use tends to decrease [17]. Intense land use causes, for example, a decline in butterfly populations and changes in butterfly community, mainly due to loss and/or reduction of breeding areas, as well as in the number of host plants for larval feeding [19,27,28].

In this study, fruit-feeding butterfly assemblages were sampled in three different forest habitats (forest interior, forest ecotone and anthropic edges), in both canopy and understory. Specifically, the following hypotheses were tested: i) fruit-feeding butterfly assemblages are more similar between ecotone and forest interior than to anthropic edges. It follows that the “brought low canopy” in the ecotone causes a shaded understory similar to the understory within the forest, at the same time, it has the eco-physiological characteristics typically found in the upper canopy of the forest interior [6]; ii) the stratification of the butterfly species composition varies differently between the three habitats. It follows that ecotone and forest interior should be more similar among them than to anthropic edges, which differs from a natural habitat (forest interior and forest ecotone) in both strata, due to type of vegetation and microclimatic conditions. The anthropic edge may vary severely in type of vegetation and microclimatic conditions, due to unpredictably of its transitional habitat [3,4]. In addition, and despite similarities between ecotone and interior forest, the physical proximity between strata in the ecotone may allow some species, coming from the understory, moving in and out the canopy.

## Material and methods

### Study site

The study was carried out in the Rio Doce State Park (PERD in the Portuguese abbreviation) (19°48’-19°29’S and 42°38’-42°28’W), in the municipalities of Marliéria, Timóteo and Dionísio, state of Minas Gerais, southeastern Brazil. The PERD covers an area of approximately 36,000 ha of Atlantic rainforest varying from 200 and 500 m above sea level, where the forest surrounds a complex system of about 42 lakes. These lakes were formed by the closure of the secondary valleys of the Doce river, after tectonic movements during the middle Holocene [29], around 10–8 thousand years ago. The surrounding rainforest, on the other hand, arose more recently (about 4,500 years old), substituting a more xeric ecosystem [29]. The present climate in the region is tropical seasonal (Aw, based on the Köppen classification), with a rainy season between October-April and a dry season between May-September. The average annual temperature is 21.9°C and the average annual precipitation is 1,480 mm [30,31].

### Sampling methods

Sampling of fruit-feeding butterflies occurred in three different habitats within the PERD: i) interior of the forest (hereafter called “forest interior”), at least 50 m distant from any border, with a canopy height similar to the anthropic edges (10–25 m high); ii) natural forest-lake or flooded grassland (hereafter “ecotone”), with high sun light availability resulting in the formation of a “brought low canopy” (5–15 m high) with main branches bent towards the lakes at 1–3 meters above the ground and with similar characteristics of forest canopy [6]; iii) anthropic edges (hereafter “edge”), a result of planned cut within the park, as in borders of dirt roads and facilities, with a canopy higher than the ecotone (between 10–30 m) but dominated by saplings and young trees close to the ground, right on the edge.

**Fig 1:**
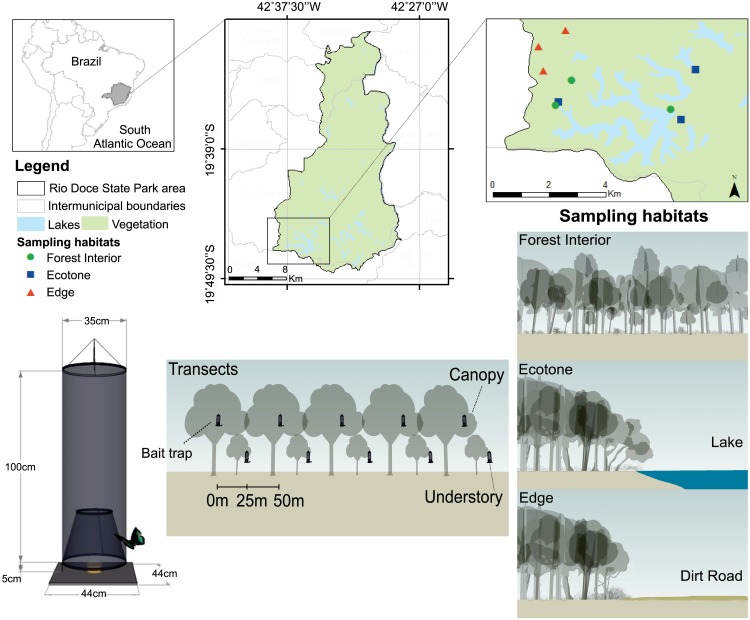
Location of the study area, Rio Doce State Park, Minas Gerais State, Brazil. Layout of the sampling design with 10 traps in each habitat (forest interior, ecotone, edge) alternating the strata (canopy, understory), in Rio Doce State Park, Brazil.

The sample design follows DeVries [32], modified after Ribeiro and Freitas [25]. Three transects of approximately 250 m in length were selected per habitat. Each habitat transect was separated by at least 1 km in distinct locations, constituting random and truly independent samples. Only two ecotone and forest interior transects were somehow in the same region, but even those were more than 300 meters apart (Fig 1). Thus, a total of nine independent transects were set: three in forest interiors, three in ecotones (from three distinct lakes) and three in edges. Each transect contained a sampling unit of 10 portable traps (Van Someren-Rydon–VSR, Fig 1) containing attractive bait (a mix of banana and sugar cane juice at a ratio 3:1, fermented for 48 hours), totaling 90 bait traps. The traps were installed 25 m apart from each other, alternating between canopy and understory, so that the canopy traps were suspended right in the upper canopy, to a distance of only 1–3 m below the canopy surface (i.e. interface between the uppermost layer of leaves and the atmosphere; [33]): 6.2–21.9 m, average of 11.14 m in the forest interior; 3.5–11.7 m, average of 7.25 m in the ecotone; 6.8–24.1 m, average of 11.44 m in the edge. The understory traps were suspended 1–1.5 m above the ground. Butterflies were sampled monthly from August 2015 to July 2016 (n = 12 months), with the traps remaining open for four consecutive days with revisions at 48 h intervals. Therefore, the total effort was 4,320 trap-days (90 traps x 4 sampling days x 12 months), a sampling effort higher than recommended by Ribeiro *et al*. [34]. During each revision, baits were replaced and all butterfly individuals captured were recorded and marked with a sequential number on the right posterior wing to avoid to overestimate butterfly abundance, thus those eventually recaptured were not counted as new individuals. Those individuals not identified in the field or that died in the traps (n = 5,958; 51.4%) were taken to the lab for later identification. At least three individuals of each butterfly species (except those with less than three individuals recorded throughout the study) were pinned and deposited at the Museu de Zoologia of the Universidade Estadual de Campinas, São Paulo, Brazil (ZUEC), as well as in the Laboratório de Ecohealph, Ecologia de Insetos de Dossel e Sucessão Natural, of the Universidade Federal de Ouro Preto, Minas Gerais, Brazil. Permits for the field studies were issued by the state authority Instituto Estadual de Florestas (IEF) and the national authority Sistema de Autorização e Informação em Biodiversidade/ Instituto Chico Mendes de Conservação da Biodiversidade (SISBIO/ ICMBio).

### Data analysis

To estimate the expected richness of fruit-feeding butterflies in the habitats (i.e., forest interior, ecotone, edge) and strata (i.e., canopy, understory) and to evaluate the representation of each sample according to the total community, the Chao 1 estimator was used. The Chao 1 estimator uses only the singletons, doubletons and the observed richness to obtain the lower bound for the expected richness [35]. Rarefaction curves were built integrating the interpolation and extrapolation (prediction) of species diversity, based on individuals, to compare species diversity among habitats and strata, using Hill numbers (q = 0, q = 1, q = 2). This unified standardization method allows the quantification and comparison of species diversity across multiple assemblages [36], even if they have unequal abundances [37]. Hill numbers are parameterized by a diversity order q, which determines the measures’ sensitivity to species relative abundances [36]. In diversity of order q = 0 all the abundances are raised to 0, in this way rare species have the same weight as abundant, so q = 0 represents species richness. In diversity of order q = 1 all the abundances are raised to 1, that is, diversity in effective species number calculated by Shannon Diversity. In diversity of order q = 2 all the abundances are raised to 2, that is, diversity in effective species number calculated by Simpson Diversity, which increases the weight of the dominant species. The package “iNEXT” in the R software was used to construct these integrated curves and the curve size was extrapolated to three times the size of the lowest observed richness to compare different samples until an estimated asymptote [36].

To examine factors affecting the distribution of fruit-feeding butterflies, Generalized Linear Models (GLMs) were used, where the abundance, species richness and subfamily-tribe abundances were used as response variables, and study habitats (forest interior, ecotone, edge), strata (canopy, understory) and the interaction between these two factors were used as explanatory variables. For this, the Poisson distribution of errors corrected for Negative Binominal distribution were used when overdispersion was verified. In addition, non-significant variables were removed until reaching the reduced model, with application of a post-hoc test to examine the difference among levels. For comparing proportion of individuals from each species between strata were used G-tests, and values were corrected using the sequential Bonferroni method [38].

In order to test what scale most contributes to the gamma diversity (γ), an analysis of additive partitioning of diversity was done using the “*vegan*” and “*betapart*” packages in R. The scales analyzed were the diversity within transects (set of five traps for each stratum) (α), the diversity distributed among transects of the same stratum and habitat (β1), between strata of the same habitat (β2) and among habitats (β3), and the total diversity of the Park (γ). Beta diversity (β1, β2 and β3) is composed of two components: turnover (i.e. replacement of some species by others) and nestedness (i.e. species found on one site represent a subset of another site), and the Jaccard index was used to separate the contribution of each process. Expected values were generated by a series of null models (with 999 simulations) and the comparisons between the observed and expected diversity were considered different when p < 0.05.

Species composition was described comparing canopy and understory in the three different habitats, using Non-Metric Multidimensional Scaling (NMDS) with Bray-Curtis similarity measure. A Permutational Multivariate Analysis of Variance (PERMANOVA) was performed to test the significance of the groupings formed by habitats and strata. The Bray-Curtis index was used to compare the similarity among and within habitats, and the coefficient of variation in the species composition within each habitat was used as a measure of biotic homogeneity. All of the statistical analyses were performed using the software R 3.4.0 [39].

For comparative purposes with previous studies, in all analyses the Nymphalidae taxonomy followed Freitas and Brown Jr [40] modified after Wahlberg *et al*. [41] (subfamilies Biblidinae, Charaxinae, Satyrinae and Nymphalinae). The subfamily Satyrinae was subdivided into three tribes (Satyrini, Morphini and Brassolini) since they are distinct in several morphological, ecological and behavioral traits (see [15]). Only a single individual of tribe Haeterini (Satyrinae) has been captured, therefore, it was excluded from the analyzes.

## Results

In total, 11,594 individuals from 98 fruit-feeding butterfly species were captured in all habitats and strata during 12 months, with Biblidinae subfamily being the most abundant (5,339 individuals, 46.05%), followed by Satyrinae (3,650 individuals, 31.48%), Charaxinae (2,495 individuals, 21.52%) and Nymphalinae (110 individuals, 0.95%) (S1 Table). Richness estimators showed that 91.9% of the total richness was sampled, which can be considered a good representation of the local assemblage (Table 1). The forest interior registered 3,151 individuals from 79 species (four exclusive species), with nine species predominantly captured in the understory, nine predominantly captured in the canopy and 48 shared between strata. The ecotone registered 4,321 individuals from 87 species (six exclusive species), with 15 species predominantly captured in the understory, only three predominantly captured in the canopy and 59 shared between strata. The edge registered 4,122 individuals from 83 species (six exclusive species), with 16 species predominantly captured in the understory, nine predominantly captured in the canopy and 57 shared between strata. The three habitats shared 70 out of the 98 recorded species (S1 Table).

**Table 1:** Abundance, richness and diversity (q0—Species richness, q1—Shannon diversity, q2—Simpson diversity) of the fruit-feeding butterflies in different habitats and strata in Rio Doce State Park, MG, Brazil.

Habitats	Abundance	Diversity			Richness estimator		
		q0	q1	q2	Chao 1	SD	Coverage %
Forest Interior							
Canopy	1408	60	19.03	12.40	70.9	7.6	84.63
Understory	1743	67	18.20	10.27	77.9	7.6	86.01
Total	3151	79	21.15	12.44	100.3	14.5	78.76
Ecotone							
Canopy	1587	73	24.06	14.33	85.5	8.5	85.38
Understory	2734	73	24.32	13.51	89.9	12.7	81.20
Total	4321	87	26.52	14.60	96.4	6.8	90.25
Edge							
Canopy	1901	71	26.99	19.07	94.1	14.8	75.45
Understory	2221	69	32.01	20.78	72.1	3.1	95.70
Total	4122	83	35.26	23.58	98.1	12.5	84.61
Total	11594	98	31.59	19.26	106.6	6.8	91.93

SD, standard deviation.

The studied habitats did not differ in fruit-feeding butterfly abundance (χ^2^ = 18.21 p = 0.093) and there was no interaction among habitats and strata (χ^2^ = 18.19 p = 0.320). On the other hand, understory had more individuals than canopy (mean ± SD: 744.2 ± 222.3 and 544 ± 174.7 respectively; χ^2^ = 22.95 p = 0.009). The tribes Morphini and Brassolini and Satyrini were more abundant in the understory (Morphini and Brassolini χ^2^ = 24.49 p < 0.001; Satyrini χ^2^ = 30.12 p < 0.001) and varied among the habitats (Morphini and Brassolini χ^2^ = 17.59 p = 0.032; Satyrini χ^2^ = 18.98 p = 0.004; Table 2). Morphini and Brassolini abundances were greater in the ecotone (n = 228 individuals) than in the forest interior and in the edge (n = 163 and 190, respectively). Satyrini abundance observed in the ecotone and in the edge (n = 1,250 and 1,069, respectively) were greater than in the forest interior (n = 750). Interaction between habitats and strata was significant only for Nymphalinae (χ^2^ = 22.02 p = 0.021), indicating greater abundance in the canopy in the forest interior and edge, but in the ecotone this subfamily was most abundant in the understory. Biblidinae and Charaxinae abundances did not vary among habitats and strata.

**Table 2:** Mean abundance for subfamilies and tribes fruit-feeding butterfly species by habitats and strata, Rio Doce State Park, Brazil.

Subfamilies/Tribe	Habitats			Strata	
	Forest Interior	Ecotone	Edge	Canopy	Understory
	Mean ± SD	Mean ± SD	Mean ± SD	Mean ± SD	Mean ± SD
Biblidinae	229.5 ± 138.8	321.5 ± 229.6	338.8 ± 38.1	285.4 ± 143.3	307.8 ± 174.0
Charaxinae	138.8 ± 73.4	146.3 ± 36.8	130.7 ± 50.9	158.4 ± 45.3	118.8 ± 54.6
Morphini and Brassolini	27.17 ± 25.1^b^	38.0 ± 33.5 ^a^	31.7 ± 27.1 ^b^	7.3 ± 4.0 ^a^	57.2 ± 13.4 ^b^
Nymphalinae	4.7 ± 2.3	6.0 ± 3.4	7.7 ± 3.6	5.9 ± 4.1	6.3 ± 2.2
Satyrini	125.0 ± 79.0 ^b^	208.3 ± 110.5 ^a^	178.2 ± 112.2 ^a^	86.9 ± 36.3 ^a^	254.1 ± 70.9 ^b^

SD, standard deviation. Different letters in front of mean indicate significant differences based on Tukey tests.

**Fig 2:**
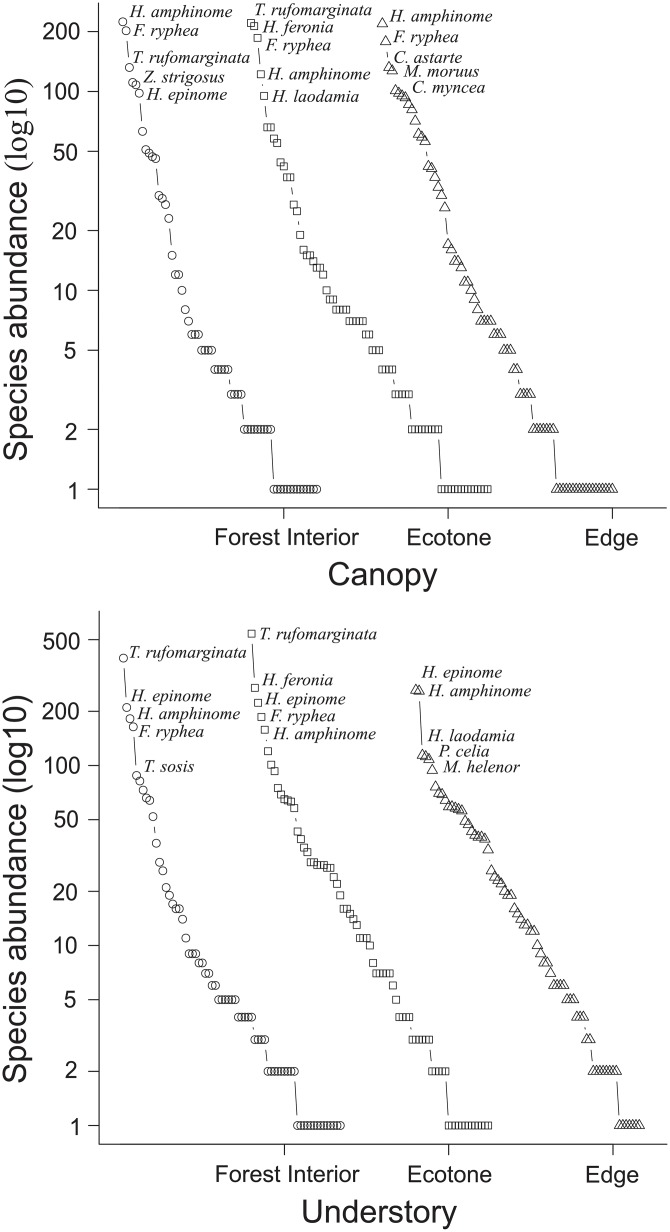
Rank-abundance distribution of fruit-feeding butterflies by habitats and strata (canopy above; understory below) in Rio Doce State Park, Brazil. The symbols represent the habitats: forest interior (circle), ecotone (square) and edge (triangle).

The species rank abundance showed a similar distribution among strata and habitats, with a predominance of rare species (Fig 2). In the canopy, 54.5% of the species (n = 48), and in the understory, 42.5% (n = 37) were represented by 10 or fewer individuals. The contributions of singletons and doubletons (species represented by one and two individuals, respectively) were 20.4% in the canopy and 21.8% in the understory (n = 18 and 19 species, respectively). In the forest interior, the most abundant species in the canopy was *Hamadryas amphinome* (n = 224 individuals; 15.9%) and in the understory, *Taygetis rufomarginata* (n = 395 individuals; 22.7%). In the ecotone, *T*. *rufomarginata* was the most abundant species in both strata, with 218 individuals in the canopy (15.5%) and 543 in the understory (19.7%). In the edge, the most abundant species in the canopy was *H*. *amphinome* (219 individuals; 11.5%) and in the understory, *H*. *epinome* (262 individuals; 11.8%). Among the five most common butterfly species, some were recorded in more than one stratum and habitat, such as *T*. *rufomarginata*, *H*. *amphinome*, *Fountainea ryphea*, *H*. *epinome* and *H*. *laodamia*. From these more common species, only *H*. *amphinome* occurred in all strata and habitats.

**Fig 3:**
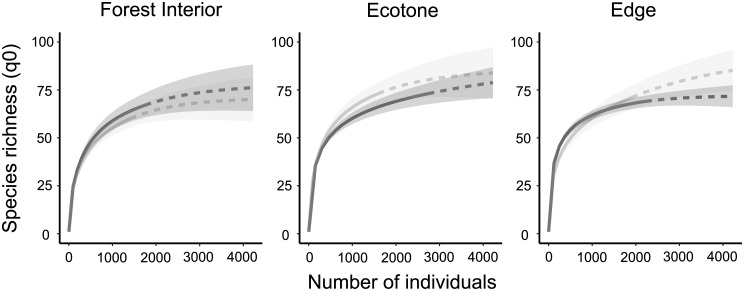
Interpolation (solid line) and extrapolation (dashed line) curves with species richness (q = 0) of fruit-feeding butterflies in Rio Doce State Park, Brazil. Shaded area represents the standard error–SE; light grey lines represent the canopy and dark grey lines the understory.

**Fig 4:**
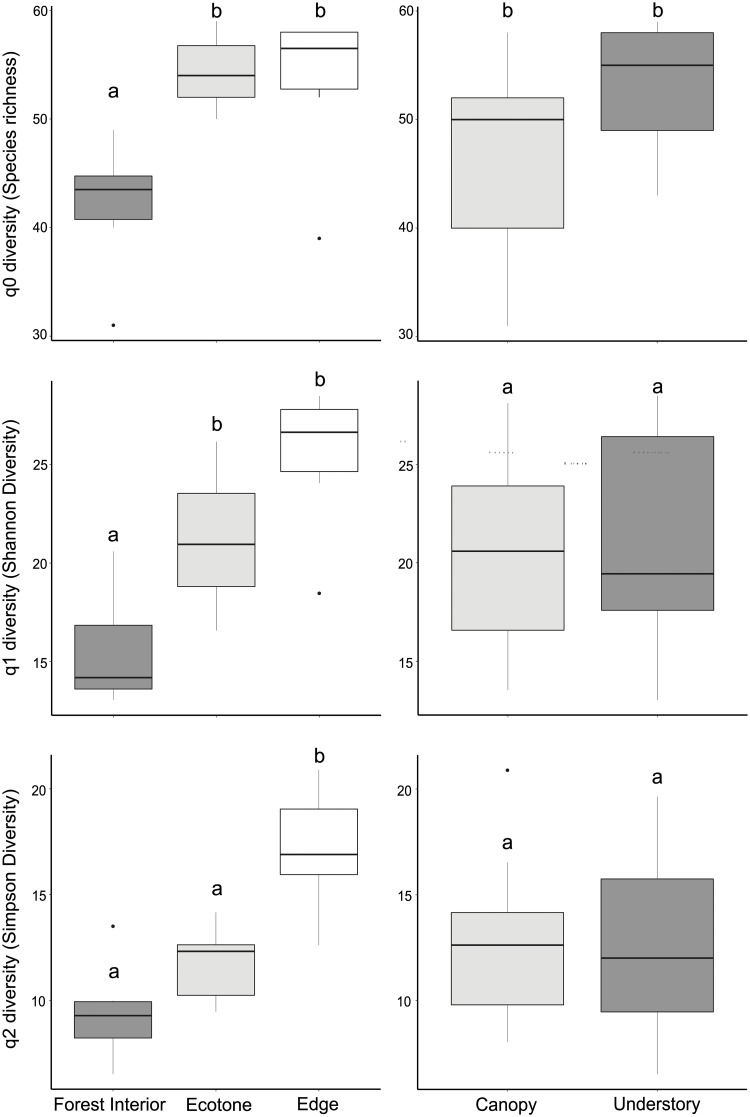
Hill’s diversity (q0—Species richness, q1—Shannon diversity, q2—Simpson diversity) of fruit-feeding butterfly species by habitats (left) and strata (right), Rio Doce State Park, Brazil. The lines represent the first and four quartiles, the box represents the second and third quartiles and the line within the box represents the median. Different letters above boxplot indicate significant differences based on Tukey tests. The points outside of the boxplot represent atypical data.

Despite the understory having a greater abundance of fruit-feeding butterflies, there was almost the same number of species in both vertical strata, and the rarefaction and extrapolation curves reveal that the species richness (q = 0) did not vary between strata (Fig 3). However, mean species richness differed among habitats, being lower in the forest interior, without difference between ecotone and edge (q = 0: χ^2^ = 8.57 p = 0.003) (Fig 4). The same can be observed for species diversity (q = 1) and species dominance (q = 2), no variation between strata (q = 1: F = 23.08 p = 0.601; q = 2: F = 0.29 p = 0.601) and differences among habitats (q = 1: F = 8.53 p < 0.001; q = 2: F = 14.06 p < 0.001) (Fig 4). The diversity (q1) of fruit-feeding butterflies was also lower in the forest interior, without difference between ecotone and edge. Meanwhile, species dominance (q = 2) was higher in the edge, without difference between ecotone and forest interior. Additionally, there was also no interaction between the habitats and strata (q = 1: F = 0.66 p = 0.532; q = 2: F = 0.64 p = 0.543).

The partitioning of species diversity analysis indicated that the average diversity among transects (α) was responsible for 50.9% of the total diversity, lower than expected at random (63.2% p = 0.001) (Figure in S5 Fig). The diversities among transects in the same stratum and habitat (β1), between strata of the same habitat (β2) and among habitats (β3) were higher than expected at random (β1 19.3%, expected 15.4%, p = 0.001; β2 14.4%, expected 8.5%, p = 0.001; β3 15.3%, expected 12.9%, p = 0.003). Thus, β1 had the greatest contribution to the β diversity of fruit-feeding butterflies, followed by β3 and β2. The decomposition into β1, β2 and β3 allowed us to verify that turnover was the main process responsible for the β diversity compared to transects (80.3%), strata (84.6%) and habitats (76.5%), while the nestedness processes explained only 19.7%, 15.4% and 24.5% respectively.

**Fig 5:**
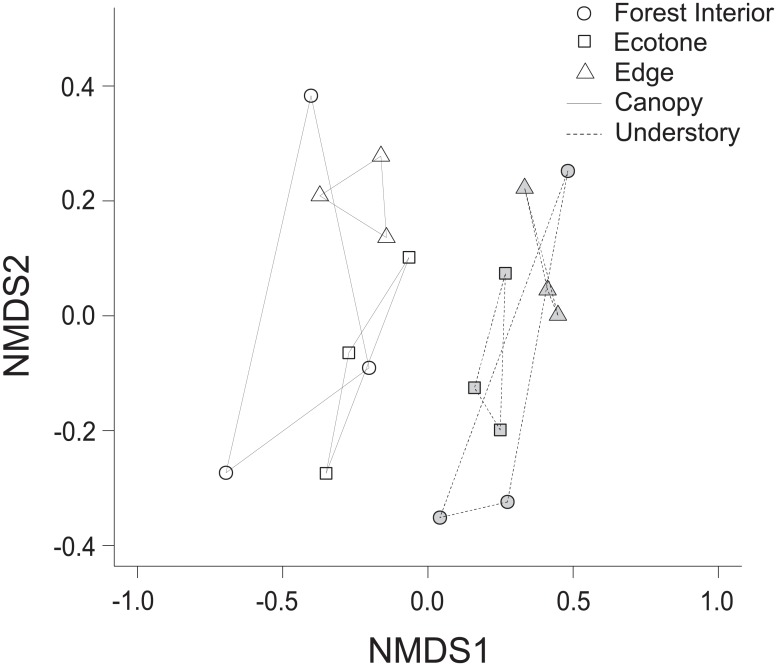
Non-metric multidimensional scaling ordination based on the composition of fruit-feeding butterfly species within habitats and strata (Bray-Curtis similarity; stress = 0.12; PERMANOVA: Strata F = 7.53, R = 0.28, p = 0.001; habitats F = 2.82, R = 0.21, p = 0.001), Rio Doce State Park, Brazil. The symbols represent the habitats: forest interior (circle), ecotone (square) and edge (triangle); and colour represents the strata: canopy (write) and understory (grey).

Species composition was distinct between canopy and understory in all habitats (stress = 0.12; PERMANOVA: F = 7.53, R = 0.28, p = 0.001) and, further, among habitats (PERMANOVA: F = 2.82, R = 0.21, p = 0.001) (Fig 5). Besides habitats differed among them, the patterns of the species distribution varied differently within each habitat. The variation in species occurrence among transects was greater in the forest interior (19.2%) than ecotone (11.7%) and also greater in the forest interior than in the edge (3.2%), which had quite homogenous samples.

## Discussion

### Butterfly diversity

The sampled fruit-feeding butterfly species richness (98 species) in the State Park of Rio Doce (PERD) was equivalent to those of other areas in the Atlantic rainforest, where species richness varies from 90 to 120 species [42]. However, in the present study, species richness was not different between strata, while in most previous studies the understory was richer than the canopy [16,22,23,32,43,44]. Still, in one study in the Amazonian forest [25] and another in the Atlantic Forest [45], the opposite was recorded, with greater fruit-feeding butterfly species richness in the canopy than in the understory. Given these idiosyncrasies one must be cautious in trying to identify a mechanism behind patterns that appear from a limited number of studies in the Atlantic Forest.

The present results showed higher abundance in the understory as found in other tropical forests [16,22,23,32,44]. A distinct pattern was found in a study carried out in an area of montane Atlantic Forest where higher abundances of fruit-feeding butterflies were observed in the canopy [45]. In that case, the greater abundance in the canopy was explained by the difference in the temperature between strata (due to a combination of altitude and latitude). The canopy of montane forests maintains higher temperatures throughout the year even in the colder months, favoring the butterfly activity in this stratum through the year [45]. In the PERD, the absence of a cold season provides high temperatures in both strata throughout the year, allowing high butterfly activity in the shaded understory all year round.

### Effects of vertical stratification for species distribution

The stratification of abundance varied by taxon. Groups known to be dominant in the canopy, such as Biblidinae and Charaxinae, did not differ between strata. Morphini and Brassolini and Satyrini, which commonly occupy lower strata, were more abundant in the understory, even in the ecotone where the brought low canopy was observed. Additionally, only Nymphalinae showed an interaction between habitat and strata, being more abundant in the ecotone understory while in the other habitats it appears to be more abundant in the canopy. This indicates that the brought low canopy in the ecotone allows typical canopy species to also occupy the understory.

Considering the vertical dimension among habitats, although the ecotone canopy is lower than the canopy of forest interior and edge, the results with fruit-feeding butterflies indicated that it still presents stratification as in other habitats. The ecotone canopy is a smooth continuation of the upper foliage surface from the interior towards the branches bent towards the lake [6]. The butterfly species that commonly dominate the canopy of the forest interior are also present in the ecotone canopy. This shows that even though it is lower, the brought low canopy in the ecotone maintains the characteristics of an upper canopy of the forest interior. Other studies in forest-lake ecotones in the PERD with other taxa (*Azteca* ant genus and galls) have also demonstrated that the ecotone maintains distribution patterns typical of upper canopy [8,11–13], being a continuation of the upper canopy and differing from the understory from the forest interior. However, the present study showed that fruit-feeding butterflies maintain the vertical stratification even in the ecotone, presenting distinct species composition between strata. On the other hand, the brought low canopy in the ecotone may allow opportunistic exploitation of its resources, as predicted. Some butterfly species such as *Taygetis rufomarginata* is a good example of this; in the present study, this species dominate the understory of the forest interior but occurred in both strata in the ecotone.

### Effects of habitat type for species distribution

It is largely known that transitions (both natural and anthropic) are usually richer and more diverse than adjacent habitats, since they combine characteristics of the two nearby environments [5] (in the present study, a forest and an open habitat). However, there are few studies comparing fruit-feeding butterfly communities between natural and anthropic transitions [17,20,24–26]. A meta-analysis using ground beetles (Coleoptera: Carabidae) showed that different edge maintenance processes (natural or anthropic) reflected in the diversity and assemblage composition of inhabitants [46]. Forest edges maintained by natural processes had higher species richness forest interiors, while edges with continued anthropic influence did not [46]. By comparing two distinct forests studied at different times, DeVries *et al*. [32] anticipated a pattern, showing that natural transitions presented lower abundance, species richness and diversity of fruit-feeding butterflies than anthropic edges, which is consistent with findings that disturbance has a positive effect on abundance and richness of butterflies (as largely known, see [20,32,47]). Nevertheless, the present study is the first to investigate with direct comparisons whether the natural transitions (forest ecotones) differ from anthropic transitions (forest edges) within a same landscape mosaic. The present study clearly indicated that both, species richness and species diversity of fruit-feeding butterflies were higher in the transition habitats (ecotone and edge) than in the forest interior, and transitional habitats did not differ markedly from each other, in contrast to the findings of DeVries *et al*. [32]. However, the results of the present study also showed that species composition and species dominance are distinct between ecotones and edges. Therefore, the disturbance origin directly affects which species will be present, as well as the community dynamics.

When comparing some of the most abundant butterfly species among habitats, several idiosyncratic responses were observed. *Taygetis rufomarginata* (the most abundant in the ecotone and the forest interior) had low abundance in the edge. This result corroborated the pattern described by Uehara-Prado *et al*. [20] that showed that large Satyrinae species prefer shaded habitats. Other species commonly recorded in edge, such as *Hamadryas amphinome* and *H*. *epinome* (Biblidinae), are also among the five most abundant species in both ecotone and forest interior. These two species are described as common in open or disturbed habitats [20], as well as *H*. *laodamia* (a species common in the edge understory and the ecotone canopy), indicating that species commonly abundant in the edge may find favorable conditions to establish themselves also in the ecotone.

The partitioning of diversity analysis showed that the β diversity among transects of the same stratum and habitat (β1) was the one that contributed most to the total fruit-feeding butterfly diversity. This indicates that transects were mainly responsible for adding new butterfly species. Therefore, the mere spatial spreading of the sampling design resulted in an important β diversity driver, even more important than strata and habitats. Hence, the spatial variation of a set of characteristics (e.g., vegetation structure, host plants and microclimatic conditions) alone may regulate the permanence of species [20,48–50]. The differences in habitat characteristics and resource distribution among areas have already been indicated as a possible explanation for the high β diversity of fruit-feeding butterflies [49]. As a consequence, species turnover was the main process responsible for the structuring the butterfly community, with the predominance of replacement some species with others among transects, strata and habitats. Natural habitats provide favorable conditions and resources for the maintenance of butterfly populations, while anthropic edges change or eliminate breeding areas and areas essential for larval feeding, drives to changes in butterfly assemblages [19,27,28].

Despite the similarities observed between ecotone and edge, due to the characteristics of transitional habitats (the highest species richness and diversity of fruit-feeding butterflies), these two types of habitats were quite distinct in species composition, with the edges showing the most homogeneous assemblages. The edge is very homogeneous compared to the natural habitats (ecotone or forest interior), so losing an edge is different from losing an ecotone. The high unpredictability caused by ongoing succession and different microclimatic conditions in the edges may favor the high dominance of a few generalist species, leading to taxonomic and functional simplification. On the other hand, the large number of lakes (42, summed up an area of 11% of the 36,000 ha of the Park) results in natural ecotones with a forest that are a habitat of great relevance at a landscape scale and also highly constant and predictable, favoring so different butterfly species. In special, this constancy in ecological conditions allowed a brought low canopy that grows occupying all possible light gaps, thus, resulting in an upper canopy-type of habitat closer to the ground. A quite unique and different situation compared to the edges.

The growing importance of human-made edges and fragmented forests to conservation resulted in natural ecotones to be neglected by ecological literature. Here, the NMDS (Fig 5) was one of the analyses that indicated that ecotones were a key habitat in terms of diversity, with a unique species composition and heterogeneous assemblages. The lower height facilitates the occupation of different butterfly species as well as the eco-physiological conditions, since many species are not able to live in the extreme temperature, wind and humidity of a typical canopy. The unique brought low canopy is capable of accommodating species adapted to distinct ecological conditions, being a repository for several populations. It is also possible to extrapolate that forest-lake ecotone is a key habitat for diversity conservation because its unique species composition and dynamics spread along a very extensive area, i.e., the huge linear extension of water-forest contacts, separated in various lakes with distinct shapes and size.

This study is the first to investigate and report differences between natural and anthropic transitions in fruit-feeding assemblages in Atlantic Forest. The main findings showed that the fruit-feeding assemblages living in the ecotones show similarities with the forest interior as well as particularities that make ecotones unique and distinct of anthropic borders. Particular characteristics attributed to ecotones favor the maintenance of butterfly populations from different habitats, consolidating their importance for the biodiversity conservation in the region. More studies are needed to better define how different ecotones are from the ecosystems stablished in human generated borders and how essential they are for biodiversity conservation in each scenario.

## Supporting information

S1 Table List of the fruit-feeding butterfly species recorded in Rio Doce State Park, state of Minas Gerais, Brazil. C, samples in canopy; U, samples in understory. Bold numbers represent significant p values (after Bonferroni’s correction). * Corrected critical *p*-value: Forest Interior = 0.002, Ecotone = 0.001, Edge = 0.001.
(PDF)

S1 Fig Abundance of fruit-feeding butterfly species per habitats (above) and strata (below), Rio Doce State Park, Brazil. The lines represent the first and four quartiles, the box represents the second and third quartiles and the line within the box represents the median. Different letters above boxplot indicate significant differences based on Tukey tests. The points outside of the boxplot represent atypical data.
(TIF)

S2 Fig Abundance for subfamilies and tribes fruit-feeding butterfly species for habitats (left) and strata (right), Rio Doce State Park, Brazil. The lines represent the first and four quartiles, the box represents the second and third quartiles and the line within the box represents the median. Different letters above boxplot indicate significant differences based on Tukey tests. The points outside of the boxplot represent atypical data.
(TIF)

S3 Fig Abundance of Nymphalinae subfamily fruit-feeding butterfly species that presented interaction among habitats and strata, Rio Doce State Park, Brazil. The lines represent the first and four quartiles, the box represents the second and third quartiles and the line within the box represents the median. Different letters above boxplot indicate significant differences based on Tukey tests. The colour represents the strata: light grey canopy and dark grey understory.
(TIF)

S4 Fig Hill’s diversity (q0—Species richness, q1—Shannon diversity, q2—Simpson diversity) of fruit-feeding butterflies associated with the habitats and strata in Rio Doce State Park, Brazil. Bars represent the standard error—SE.
(TIF)

S5 Fig Spatial partitioning of fruit-feeding butterfly diversity in Rio Doce State Park, Brazil. Observed and expected diversity across multiple scales: α1 (black) = diversity within transects (set of five traps for each stratum); β1 (dark grey) = difference of diversity among transects of the same stratum and habitat; β2 (grey) = difference of diversity between strata of the same habitat; and β3 (light grey) = difference of diversity among habitats.
(TIF)

S6 Fig Cluster analyses of the fruit-feeding butterfly assemblages (Bray-Curtis similarity), Rio Doce State Park, Brazil. The numbers above the branch represent the p-values: left number is the approximately unbiased (AU) and right number is bootstrap probability (BP). The numbers below the branch represent the grouping sequence of Cluster analyses.
(TIF)
