# Supplementary material for: Equal but different: Natural ecotones are dissimilar to anthropic edges
Source: PLoS One. 2019 Mar 4;14(3):e0213008. doi: 10.1371/journal.pone.0213008 (PMC6398848; doi:10.1371/journal.pone.0213008)
Supplement: S1 Table — C, samples in canopy; U, samples in understory. Bold numbers represent significant p values (after Bonferroni’s correction). * Corrected critical p-value: Forest Interior = 0.002, Ecotone = 0.001, Edge = 0.001. (PDF) [file pone.0213008.s001.pdf]

Subfamilies/Tribes	Forest Interior				Ecotone				Edge				Total
	C	U	G-test	p*	C	U	G-test	p*	C	U	G-test	p*	
**BIBLIDINAE**	710	667	---	---	797	1132	---	---	1062	971	---	---	5339
Biblis hyperia	0	0	---	---	0	0	---	---	1	2	---	---	3
Callicore astarte	98	37	28.59	**< 0.0001**	42	33	1.08	0.2981	132	56	31.62	**< 0.0001**	398
Callicore sorana	0	0	---	---	1	0	---	---	7	2	---	---	10
Callicore texa	1	0	---	---	1	1	---	---	2	0	---	---	5
Catonephele acontius	4	9	1.97	0.1601	2	7	1.73	0.1879	1	7	---	---	30
Catonephele numilia	12	4	4.19	0.0408	19	28			26	8	10.03	0.0015	97
Diaethria clymena	10	1	8.55	0.0035	66	14	36.71	**< 0.0001**	13	6	2.64	0.1042	110
Ectima thecla	0	0	---	---	0	1	---	---	0	0	---	---	1
Epiphile sp.	0	0	---	---	1	0	---	---	0	0	---	---	1
Eunica eurota	1	0	---	---	0	0	---	---	0	0	---	---	1
Eunica maja	3	14	7.72	0.0055	14	28	---	---	4	13	5.02	0.0255	76
Eunica malvina	2	3	---	---	9	3	---	---	3	0	---	---	20
Eunica mygdonia	1	0	---	---	2	0	---	---	4	1	---	---	8
Eunica tatila	1	0	---	---	3	0	---	---	1	0	---	---	5
Haemathera pyrame	0	0	---	---	0	0	---	---	1	0	---	---	1
Hamadryas amphinome	224	182	4.35	0.0370	122	158	4.35	0.0370	219	259	3.35	0.0672	1164
Hamadryas arete	49	19	13.70	**0.0002**	13	11	13.70	**0.0002**	42	23	5.64	0.0176	157
Hamadryas chloe	0	1	---	---	1	22	23.66	**< 0.0001**	0	19	---	---	43
Hamadryas epinome	110	208	30.70	**0.0001**	64	225	95.03	**< 0.0001**	81	262	100.53	**< 0.0001**	950
Hamadryas februa	0	8	---	---	7	29	14.44	**0.0001**	5	4	---	---	53
Hamadryas feronia	29	64	13.50	**0.0002**	212	271	7.23	0.0072	61	57	0.14	0.7127	694
Hamadryas iphthime	6	1	---	---	3	1	---	---	1	2	---	---	14
Hamadryas laodamia	51	66	1.93	0.1649	94	121	3.40	0.0652	93	114	2.13	0.1441	539
Myscelia orsis	4	26	18.03	**< 0.0001**	6	101	102.10	**< 0.0001**	2	49	53.83	**< 0.0001**	188
Nica flavilla	1	6	---	---	8	27	10.89	**0.0010**	14	40	13.05	**0.0003**	96
Paulogramma pygas	46	7	32.10	**< 0.0001**	44	15	14.89	**0.0001**	178	26	127.15	**< 0.0001**	316
Pyrrhogyra neaeria	0	0	---	---	0	0	---	---	2	0	---	---	2
Temenis huebneri	30	4	22.50	**< 0.0001**	37	19	5.89	0.0152	98	8	90.22	**< 0.0001**	196
Temenis laothoe	27	7	12.56	**0.0004**	26	17	1.90	0.1683	71	13	44.06	**< 0.0001**	161

Subfamilies/Tribes	Forest Interior				Ecotone				Edge				Total
	C	U	G-test	p*	C	U	G-test	p*	C	U	G-test	p*	
**CHARAXINAE**	496	337	---	---	425	453	---	---	505	279	---	---	2495
Agrias claudina	4	0	---	---	2	0	---	---	0	1	---	---	7
Archaeoprepona amphimachus	2	5	---	---	0	5	---	---	5	10	1.70	0.1924	27
Archaeoprepona demophon	6	17	5.48	0.0192	15	27	3.48	0.0622	30	47	3.78	0.0517	142
Archaeoprepona demophoon	5	5	---	---	7	4	0.83	0.3627	14	6	3.29	0.0696	41
Archaeoprepona meander	0	1	---	---	0	1	---	---	0	0	---	---	2
Fountainea glycerium cratais	1	0	---	---	1	0	---	---	0	0	---	---	2
Fountainea ryphea	202	164	3.95	0.0468	185	187	0.01	0.9174	86	59	5.06	0.0245	883
Hypna clytemnestra	1	11	9.75	**0.0018**	4	39	33.00	**< 0.0001**	0	5	---	---	60
Memphis acidalia	15	21	1.01	0.3162	37	43	0.45	0.5021	33	43	1.32	0.2507	192
Memphis moruus	63	29	12.87	**0.0003**	59	64	0.20	0.6521	127	58	26.37	**< 0.0001**	400
Memphis xenocles	47	6	36.04	**< 0.0001**	24	7	9.86	0.0017	56	5	49.97	**< 0.0001**	145
Prepona dexamenus	1	0	---	---	1	0	---	---	1	0	---	---	3
Prepona eugenes	2	0	---	---	1	1	---	---	7	0	---	---	11
Prepona laertes	4	2	---	---	7	7	---	---	8	0	---	---	28
Prepona pseudomphale	8	2	3.86	0.0496	12	3	5.78	0.0162	6	0	---	---	31
Prepona sp.	1	0	---	---	0	0	---	---	0	0	---	---	1
Siderone galanthis	23	1	24.96	**< 0.0001**	16	1	15.96	**< 0.0001**	37	4	30.62	**< 0.0001**	82
Zaretis strigosus	111	73	7.91	0.0049	54	64	0.85	0.3570	95	41	22.04	**< 0.0001**	438
**NYMPHALINAE**	14	14	---	---	11	25	---	---	28	18	---	---	110
Colobura dirce	2	9	4.82	0.0282	1	16	15.96	**< 0.0001**	1	16	15.96	**< 0.0001**	45
Historis acheronta	5	2			7	2			10	0			26
Historis odius	7	3	1.65	0.1996	3	7	1.65	0.1996	17	2	13.55	**0.0002**	39
**SATYRINAE**	188	725	---	---	354	1124	---	---	306	953	---	---	3650
**Brassolini**	12	65	---	---	24	105	---	---	21	60	---	---	287
Caligo brasiliensis	0	0	---	---	0	2	---	---	1	6	---	---	9
Caligo illioneus	0	5	---	---	4	29	21.37	**< 0.0001**	0	15	---	---	53
Catoblepia amphirhoe	0	2	---	---	2	2	---	---	1	3	---	---	10
Catoblepia berecynthia	1	2	---	---	0	3	---	---	1	9	7.36	0.0067	16
Eryphanis automedon	6	52	41.82	**< 0.0001**	8	64	49.58	**< 0.0001**	1	12	10.97	**0.0009**	143
Opoptera aorsa	0	0	---	---	0	0	---	---	0	1	---	---	1

Subfamilies/Tribes	Forest Interior				Ecotone				Edge				Total
	C	U	G-test	p*	C	U	G-test	p*	C	U	G-test	p*	
Opsiphanes cassiae	0	0	---	---	1	1	---	---	0	0	---	---	2
Opsiphanes invirae	5	4	---	---	7	4	0.83	0.3627	15	13	0.14	0.7053	48
Opsiphanes quiteria	0	0	---	---	2	0	---	---	2	1	---	---	5
**Haeterini**	0	1	---	---	0	0	---	---	0	0	---	---	1
Pierella lamia	0	1	---	---	0	0	---	---	0	0	---	---	1
**Morphini**	2	84	---	---	6	93	---	---	1	108	---	---	294
Antirrhea archaea	0	2	---	---	0	0	---	---	0	0	---	---	2
Morpho helenor	2	82	97.55	**< 0.0001**	6	93	91.97	**< 0.0001**	1	108	139.73	**< 0.0001**	292
**Satyrini**	174	575	---	---	324	926	---	---	284	785	---	---	3068
Archeuptychia cluena	1	16	15.96	**< 0.0001**	0	3	---	---	0	6	---	---	26
Caeruleuptychia brixius	5	8	0.70	0.4033	0	0	---	---	0	2	---	---	15
Caeruleuptychia sp.1	12	1	10.97	**0.0009**	13	7	1.83	0.1764	1	0	---	---	34
Caeruleuptychia sp.2	0	0	---	---	0	0	---	---	1	0	---	---	1
Chloreuptychia arnaca	0	4	---	---	0	1	---	---	0	19	---	---	24
Chloreuptychia herseis	0	3	---	---	1	2	---	---	0	4	---	---	10
Cissia eous	1	0	---	---	2	4	---	---	3	40	37.85	**< 0.0001**	50
Cissia myncea	3	1	---	---	15	24	2.10	0.1477	101	64	8.37	0.0038	208
Cissia phronius	0	1	---	---	0	7	---	---	6	39	27.04	**< 0.0001**	53
Euptychoides castrensis	0	0	---	---	1	0	---	---	0	1	---	---	2
Hermeuptychia sp.	2	0	---	---	4	4	---	---	6	3	---	---	19
Magneuptychia lea	0	0	---	---	0	1	---	---	0	0	---	---	1
Magneuptychia libye	0	0	---	---	3	0	---	---	0	0	---	---	3
Magneuptychia sp.1	0	0	---	---	0	1	---	---	0	0	---	---	1
Pareuptychia ocirrhoe	1	5	---	---	9	58	40.01	**< 0.0001**	11	94	75.12	**< 0.0001**	178
Pareuptychia summandosa	0	1	---	---	10	35	14.71	**0.0001**	5	70	67.23	**< 0.0001**	121
Paryphthimoides grimon	0	0	---	---	5	1	---	---	0	2	---	---	8
Paryphthimoides poltys	2	2	---	---	5	13	3.68	0.0550	11	20	2.65	0.1035	53
Paryphthimoides sp.1	0	0	---	---	0	1	---	---	1	0	---	---	2
Pharneuptychia sp.1	0	1	---	---	1	2	---	---	3	5	---	---	12
Pseudodebis celia	0	0	---	---	1	0	---	---	59	112	16.70	**< 0.0001**	172
Pseudodebis euptychidia	0	16	---	---	0	28	---	---	7	59	46.85	**< 0.0001**	110
Splendeuptychia doxes	4	3	---	---	8	8	---	---	3	1	---	---	27

Subfamilies/Tribes	Forest Interior				Ecotone				Edge				Total
	C	U	G-test	p*	C	U	G-test	p*	C	U	G-test	p*	
Taygetina kerea	0	4	---	---	0	1	---	---	0	0	---	---	5
Taygetis fulginia	0	0	---	---	0	0	---	---	0	1	---	---	1
Taygetis laches	0	9	---	---	8	69	55.38	< **0.0001**	1	69	86.56	< **0.0001**	156
Taygetis leuctra	1	5	---	---	1	3			0	0	---	---	10
Taygetis mermeria	2	2	---	---	3	11	4.86	0.0275	2	14	10.12	0.0015	34
Taygetis rufomarginata	132	395	137.33	< **0.0001**	218	543	143.36	< **0.0001**	41	76	10.63	**0.0011**	1405
Taygetis sosis	3	88	99.78	< **0.0001**	1	75	94.71	< **0.0001**	1	24	26.26	< **0.0001**	192
Taygetis virgilia	0	5	---	---	0	10	---	---	2	34	34.46	< **0.0001**	51
Unidentified	0	1	---	---	4	0	---	---	2	2	---	---	9
Yphthimoides affinis	0	2	---	---	2	11	6.86	0.0088	7	22	8.15	0.0043	44
Yphthimoides renata	3	1	---	---	5	3	---	---	9	2	4.82	0.0282	23
Zischkaia pacarus	0	1	---	---	2	0	---	---	1	0	---	---	4
Zischkaia saundersii	2	0	---	---	2	0	---	---	0	0	---	---	4
**Total of individuals**	**1408**	**1743**			**1587**	**2734**			**1901**	**2221**			**11594**

C, samples in canopy; U, samples in understory. Bold numbers represent significant p values (after Bonferroni's correction). * Corrected critical *p*-value: Forest Interior = 0.002, Ecotone = 0.001, Edge = 0.001.
