# Supplementary figures and images for: Equal but different: Natural ecotones are dissimilar to anthropic edges
Source: PLoS One. 2019 Mar 4;14(3):e0213008. doi: 10.1371/journal.pone.0213008 (PMC6398848; doi:10.1371/journal.pone.0213008)

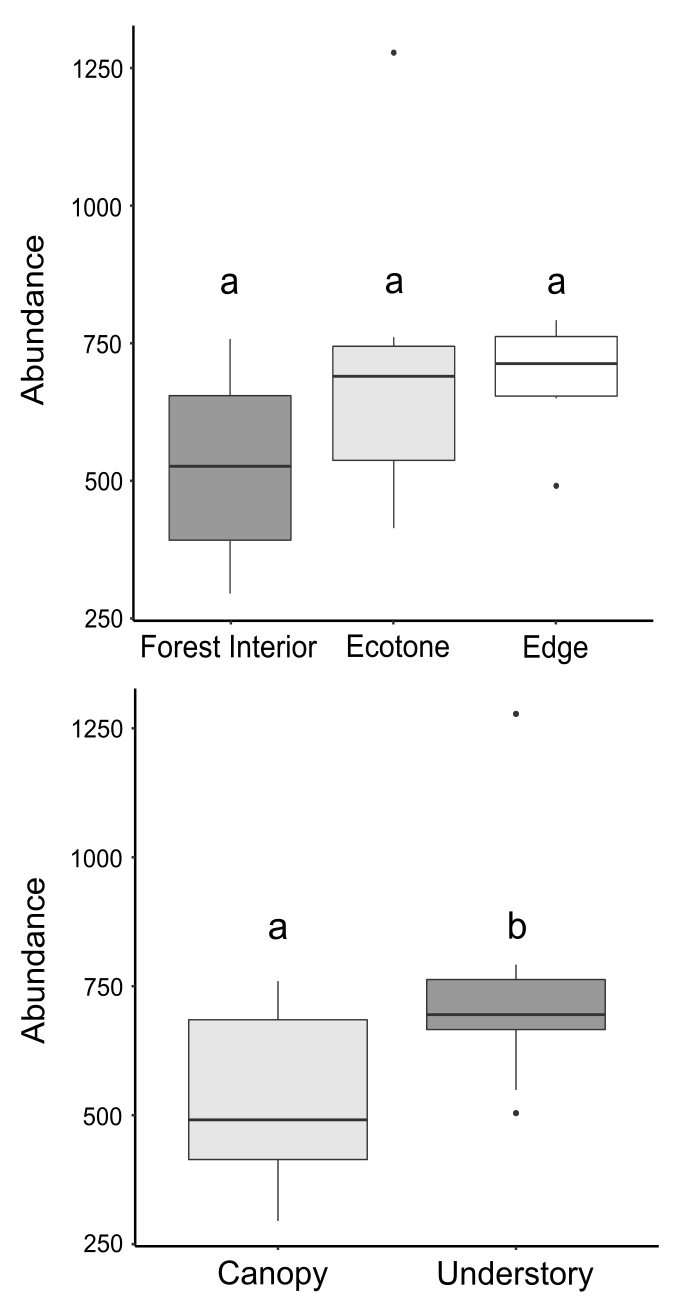

Supplement: S1 Fig — The lines represent the first and four quartiles, the box represents the second and third quartiles and the line within the box represents the median. Different letters above boxplot indicate significant differences based on Tukey tests. The points outside of the boxplot represent atypical data. (TIF) [file pone.0213008.s002.tif]

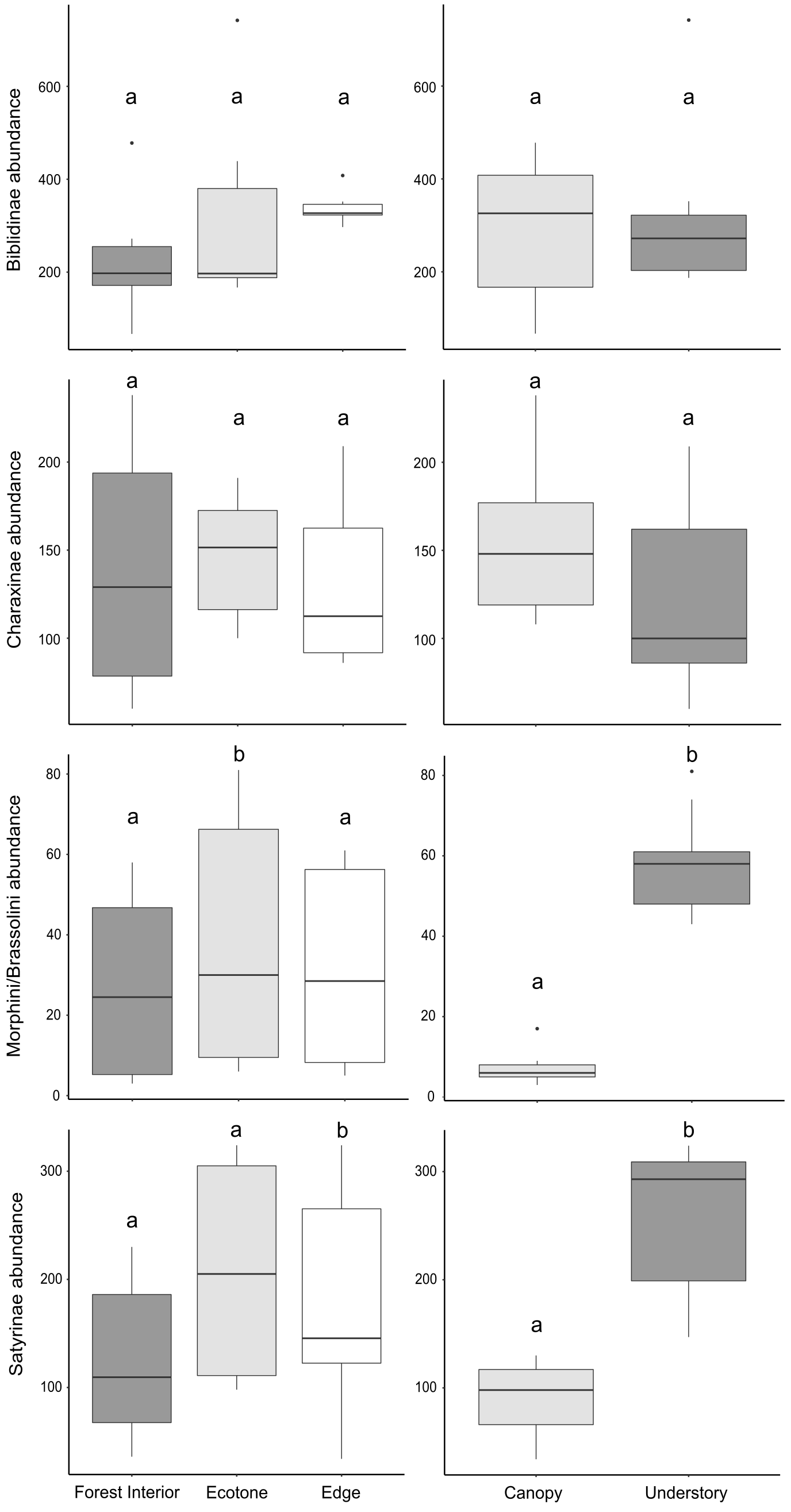

Supplement: S2 Fig — The lines represent the first and four quartiles, the box represents the second and third quartiles and the line within the box represents the median. Different letters above boxplot indicate significant differences based on Tukey tests. The points outside of the boxplot represent atypical data. (TIF) [file pone.0213008.s003.tif]

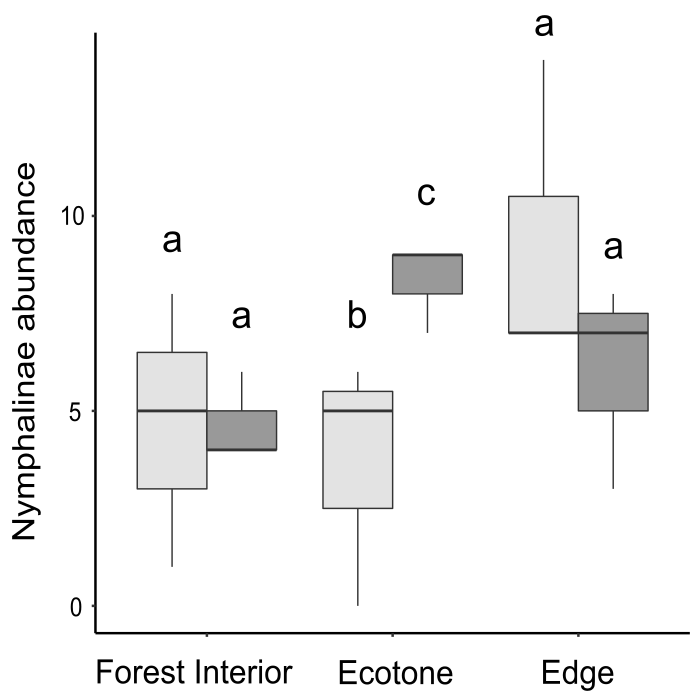

Supplement: S3 Fig — The lines represent the first and four quartiles, the box represents the second and third quartiles and the line within the box represents the median. Different letters above boxplot indicate significant differences based on Tukey tests. The colour represents the strata: light grey canopy and dark grey understory. (TIF) [file pone.0213008.s004.tif]

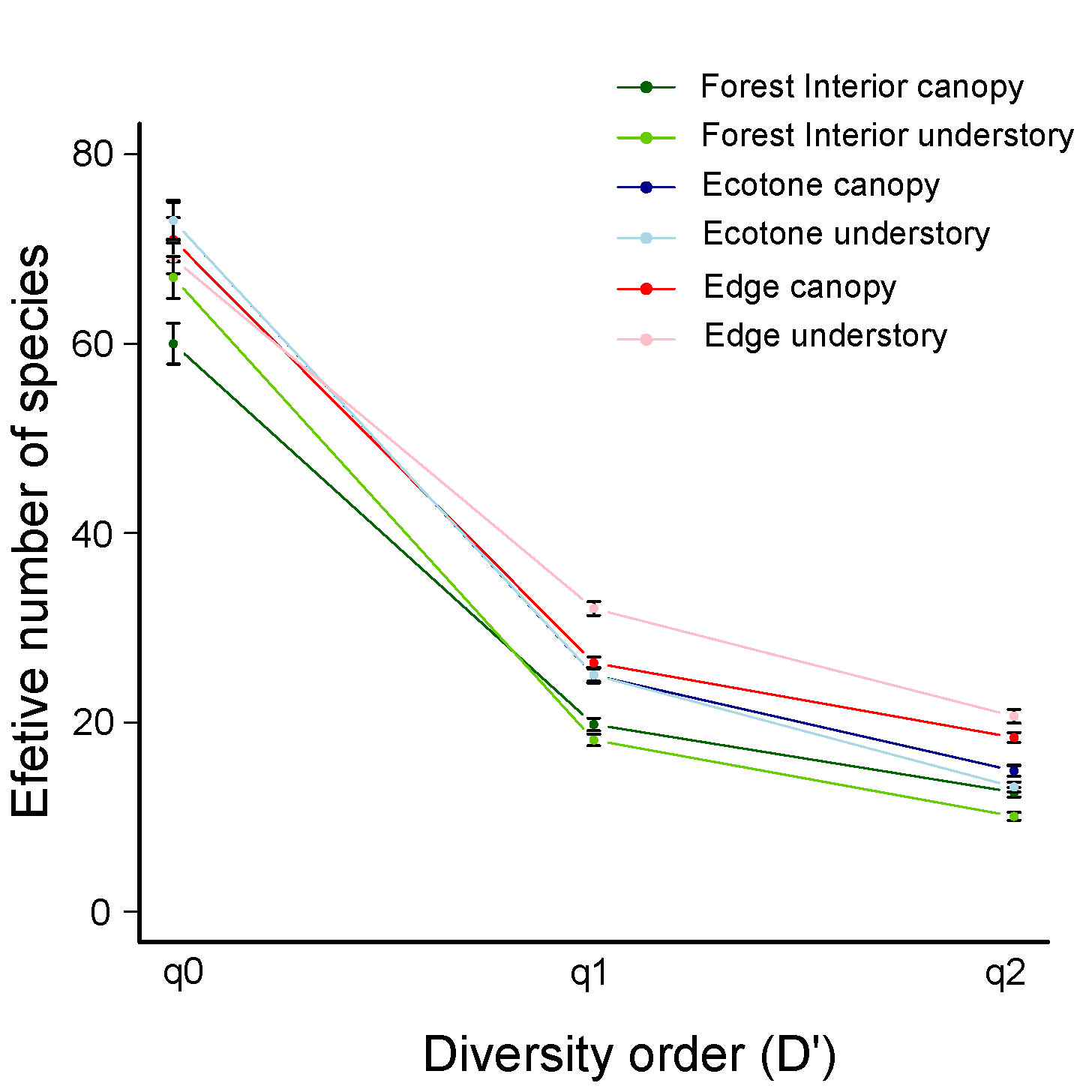

Supplement: S4 Fig — Bars represent the standard error—SE. (TIF) [file pone.0213008.s005.tif]

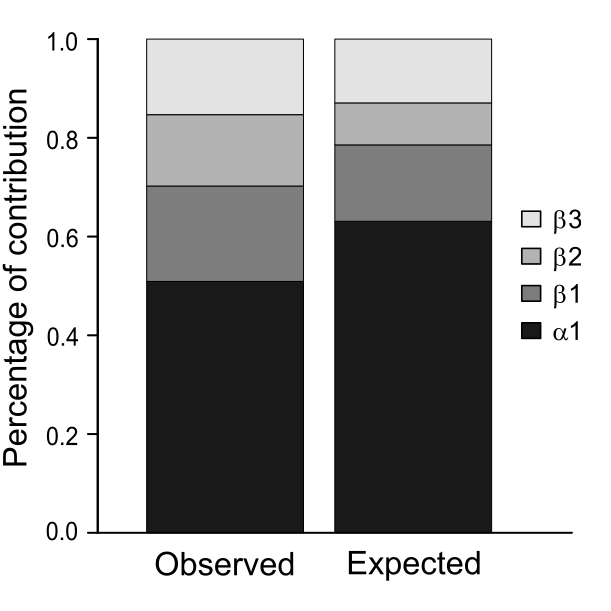

Supplement: S5 Fig — Observed and expected diversity across multiple scales: α1 (black) = diversity within transects (set of five traps for each stratum); β1 (dark grey) = difference of diversity among transects of the same stratum and habitat; β2 (grey) = difference of diversity between strata of the same habitat; and β3 (light grey) = difference of diversity among habitats. (TIF) [file pone.0213008.s006.tif]

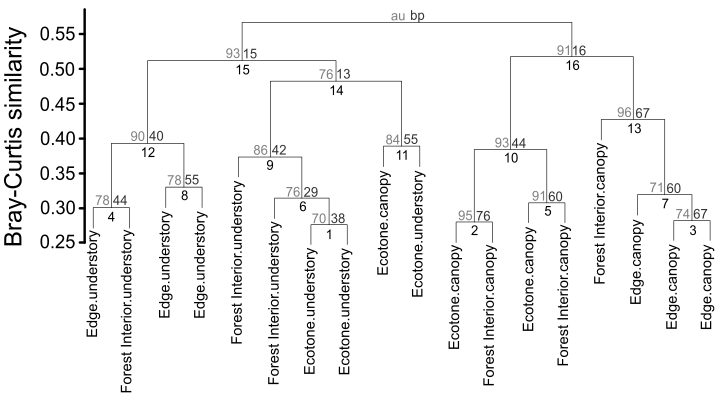

Supplement: S6 Fig — The numbers above the branch represent the p-values: left number is the approximately unbiased (AU) and right number is bootstrap probability (BP). The numbers below the branch represent the grouping sequence of Cluster analyses. (TIF) [file pone.0213008.s007.tif]
